# APOE4‐Linked Gut Microbiome Alterations and Alzheimer's Risk via the Gut‐Brain Axis

**DOI:** 10.1002/alz70856_103718

**Published:** 2025-12-24

**Authors:** Chenyu Liu, Liangliang Zhang

**Affiliations:** ^1^ Case Western Reserve Univeristy, Cleveland, OH, USA

## Abstract

**Background:**

The gut–brain axis hypothesis proposes a bidirectional communication network between the gut microbiome and the central nervous system, shaping neuroinflammatory processes linked to Alzheimer's disease (AD). Although the APOE4 allele is the strongest genetic risk factor for AD—raising the likelihood of disease by two‐ to three‐fold with even one copy—its association with the gut microbiome remains underexplored. This gap limits our full understanding of the pathways contributing to AD.

**Method:**

We investigated the relationship between APOE4 status and gut microbiome composition in 114 healthy participants (average age: 77, 57% women). Stool samples underwent shotgun metagenomic sequencing. Rigorous quality control steps removed low‐quality reads and human DNA contaminants. We performed taxonomic profiling and applied rarefaction to normalize sequencing depth. Alpha diversity (richness and evenness) and beta diversity (unweighted UniFrac‐based principal coordinates analysis) were assessed. We then used permutational multivariate analysis of variance, adjusting for demographic and clinical variables, to identify group differences. Differential taxonomic analysis pinpointed bacterial taxa enriched in APOE4 carriers versus non‐carriers.

**Result:**

Alpha diversity metrics did not differ significantly between APOE4 carriers and non‐carriers at the species level (*p* = 0.070). However, beta diversity analysis showed significant differences in overall community composition after adjusted by the covariates (*p* = 0.003), and APOE4 carrier status remained significant in PERMANOVA (*p* = 0.039). Furthermore, subgroup analysis of APOE4 genotypes (2/4, 3/4, 4/4) also revealed significant compositional differences (*p* = 0.030). Differential taxonomic analysis identified 21 species enriched in APOE4 carriers and 20 species enriched in non‐carriers. Among non‐carriers, *Alistipes finegoldii* (*p* = 0.035) and *Odoribacter splanchnicus* (*p* = 0.024) were more abundant. These species are involved in metabolic pathways related to short‐chain fatty acid production, which can have anti‐inflammatory effects. Their presence suggests a protective gut microbiome–mediated mechanism in individuals without the APOE4 allele.

**Conclusion:**

Our findings suggest that APOE4 carriers have distinct gut microbiome patterns that may heighten the risk of neuroinflammation through the gut–brain axis, potentially contributing to AD onset or progression. These results highlight the interplay between genetic risk factors and gut microbial communities. They also underscore the potential for microbiome‐targeted interventions to reduce AD risk in genetically susceptible individuals.

